# Three-Month Results of Brolucizumab Intravitreal Therapy in Patients with Wet Age-Related Macular Degeneration

**DOI:** 10.3390/ijerph18168450

**Published:** 2021-08-10

**Authors:** Katarzyna Michalska-Małecka, Dorota Śpiewak, Dorota Luksa

**Affiliations:** 1 University Clinical Center, University Hospital Medical University of Silesia, 40-514 Katowice, Poland; dorota.spiewak7@gmail.com (D.Ś.); dorota.luksa@gmail.com (D.L.); 2 Department of Ophthalmology, Medical University of Silesia, 40-514 Katowice, Poland

**Keywords:** brolucizumab, wet age-related macular degeneration, anti-VEGF, intravitreal injections

## Abstract

The purpose of the study was to evaluate changes in best corrected visual acuity, central retinal thickness, area and flow in the neovascular membrane and to compare therapeutic outcomes from baseline in patients who received three doses of Beovu (brolucizumab) at three-month follow-up. Material and methods: A prospective observational study conducted at the Prof. K. Gibiński University Clinical Center of the Medical University of Silesia in Katowice. Eight patients with exudative form of age-related macular degeneration (AMD) were observed. Results: The mean best corrected visual acuity (BCVA) outcome increased with each subsequent visit. The mean central retinal thickness (CRT) result also improved (decreased) with each subsequent visit, except for the last measurement. A statistically significant change in neovascular membrane area was observed after the first injection. In further treatment, the membrane area underwent changes that were not statistically significant. A statistically significant change in neovascular membrane flow was demonstrated after the first and second injections. Discussion: Our study confirmed the efficacy of brolucizumab in the treatment of patients with exudative AMD in terms of improvements in best corrected visual acuity (BCVA), central retinal thickness (CRT), neovascular membrane area, and neovascular membrane flow area.

## 1. Introduction

Age-related macular degeneration (AMD) is a chronic, progressive degenerative disease of the macula that mainly affects the elderly population and leads to impaired central vision [1]. AMD is the third cause of blindness in the world, after cataracts and glaucoma, while in developed countries it is the main cause [2]. There are dry and exudative forms of AMD. The pathomechanism of exudative AMD is complex and not fully understood. Retinal and choroidal hypoxia may cause dysregulation of vascular endothelial growth factor (VEGF) production by the retinal pigment epithelium (RPE). As a result, growth of abnormal vessels is promoted, mainly from the choroid, and much less frequently from the retina [3]. Regular intravitreal administration of anti-VEGF agents, as a standard treatment for exudative AMD, significantly improves patient prognosis [4]. In 2004, following Food and Drug Administration (FDA) approval, a formulation containing pegaptanib (Macugen^®^, Eyetech Pharmaceuticals, Inc-Pfizer Inc.) was administered, followed by ranibizumab (Lucentis^®^, Genentech Inc., South San Francisco) in 2006, and aflibercept (Eylea^®^, Regeneron Pharmaceuticals and Bayer HealthCare) in 2012 [5]. The latest drug in this group, launched in Europe in February 2020, is brolucizumab (Beovu^®^, Novartis).

The medicinal product Beovu (brolucizumab) is a humanized single-chain fragment of monoclonal antibody Fv (scFv) produced by recombinant DNA in *Escherichia coli* cells. The recommended dose is 6 mg of brolucizumab (0.05 mL solution) administered by injection into the vitreous body every four weeks for the first 3 doses—the saturation phase. Thereafter, the doctor individually determines the intervals between subsequent injections, depending on the activity of the disease. Acceptable intervals between successive injections are 3 months, which is a breakthrough in the scheme of administration of anti-VEGF preparations. This is the first anti-VEGF drug with proven clinical efficacy that provides the option of giving injections every 3 months immediately after the saturation phase [6].

The purpose of this study was to evaluate changes in best corrected visual acuity, central retinal thickness, area, and neovascular membrane flow and to compare therapeutic outcomes from baseline in patients who received three doses of Beovu (brolucizumab) at 3-month follow-up.

## 2. Material and Methods

A prospective observational study was conducted at the Prof. K. Gibinski University Clinical Center of the Silesian Medical University in Katowice from July 2020. Patients were qualified for treatment with brolucizumab according to the criteria of the National Health Fund Drug Program for exudative AMD. According to the criteria, inclusion in the Drug Program "Treatment of neovascular (exudative) form of age-related macular degeneration (AMD)" requires all the following conditions: (1) presence of active (primary or secondary), classic, occult or mixed subretinal neovascularization (CNV), occupying more than 50% of the AMD lesion, confirmed by OCT (optical coherence tomography) and fluorescein angiography or angio-OCT; (2) age over 45. years of age; (3) lesion size < 12 DA (12 optic nerve disc areas); (4) best corrected visual acuity (BCVA) in the treated eye from 0.2 to 0.8 as determined by the Snellen chart (ETDRS equivalent: from 50 to 80 letters); (5) patient consent for intravitreal injections; (6) no predominant geographic atrophy; (7) no predominant haemorrhage. Exclusion criteria are: (1) hypersensitivity to the drug or to any of the excipients, (2) active infection of the eye or its surroundings, active severe intraocular inflammation, (3) pregnancy or breastfeeding, (4) occurrence of drug-related adverse reactions that prevent further use, (5) hematogenous retinal detachment or macular hole of the 3rd or 4th stage (6) progression of the disease, defined as: deterioration of the best corrected visual acuity (BCVA) to a value <0.2 according to the Snellen table (ETDRS equivalent: 50 letters), lasting longer than 2 months or permanent damage to the foveal structure, which makes stabilization or functional improvement impossible.

The study obtained a positive opinion of the Bioethics Committee by Resolution No. KNW/0022/KB1/38//III/15/16 of 29 November 2016 of the Bioethics Committee of the Medical University of Silesia in Katowice. All patients provided written informed consent and this study was conducted in accordance with The Declaration of Helsinki. The study group includes eight patients (8 eyes): three women and five men, ranging in age from 59 to 84 years. The mean age of the patients is 71.25 years. The patients were qualified for treatment with Beovu in July 2020. None of the patients had previously received anti-VEGF injections into the vitreous chamber of the study eye. They received three saturating injections of the drug at a dose of 6 mg intravitreal, each time at monthly intervals, starting in July 2020. This article refers to results from this study period. The study is continuing, and further results will be reported at a later time, after the required data have been collected.

Assessment of the fundus was performed using indirect slit lamp viewing. The best corrected visual acuity was assessed using ETDRS illuminated charts. Best corrected visual acuity (BCVA), central retinal thickness (CRT), and area and flow through the neovascular membrane were assessed on the day of each injection and at the follow-up visit on the seventh day after the injection, corresponding to six control points in each patient. The RTVue XR OCT Avanti system was used to evaluate retinal parameters. One and the same doctor measured the area of the neovascular membrane each time by manually outlining its borders.

## 3. Statistical Analysis

The conformity of the distributions of the studied variables to the normal distribution was checked (Shapiro-Wilk test). Based on the results obtained, a decision was made to use the appropriate statistical test. For variables whose distribution did not conform to normal distribution, the Wilcoxon test was used for comparisons between two consecutive measurements, and for variables whose distribution conformed to normal distribution, the Student’s *t*-test for dependent samples was performed.

## 4. Results

Eight eyes of eight patients who received three monthly injections of brolucizumab into the vitreous chamber of one eye were analyzed. Baseline characteristics of the group in terms of age, sex, and baseline visual acuity, retinal thickness, neovascular membrane area, and neovascular membrane flow are presented in Table 1. The main objective of the study was to evaluate changes in best corrected visual acuity, central retinal thickness, area, and flow through the neovascular membrane and to compare the results with baseline values. Measurements were made on the day of each injection (visit 1, 3, 5, which corresponds to the individual days of the study: 1, 31, 61) and seven days after intravitreal injection (visit 2, 4, 6, which corresponds to the individual days of the study: 8, 38, 68), respectively.

**Figure 1:**
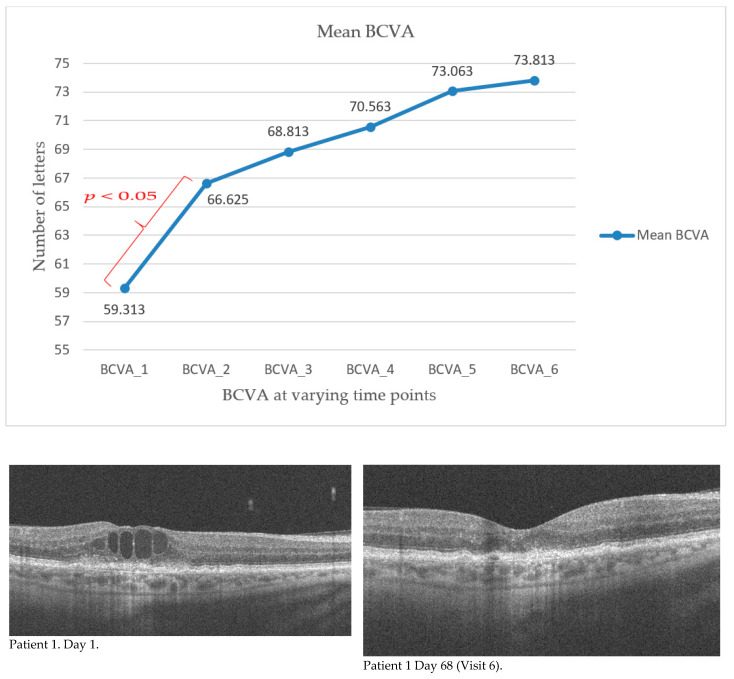

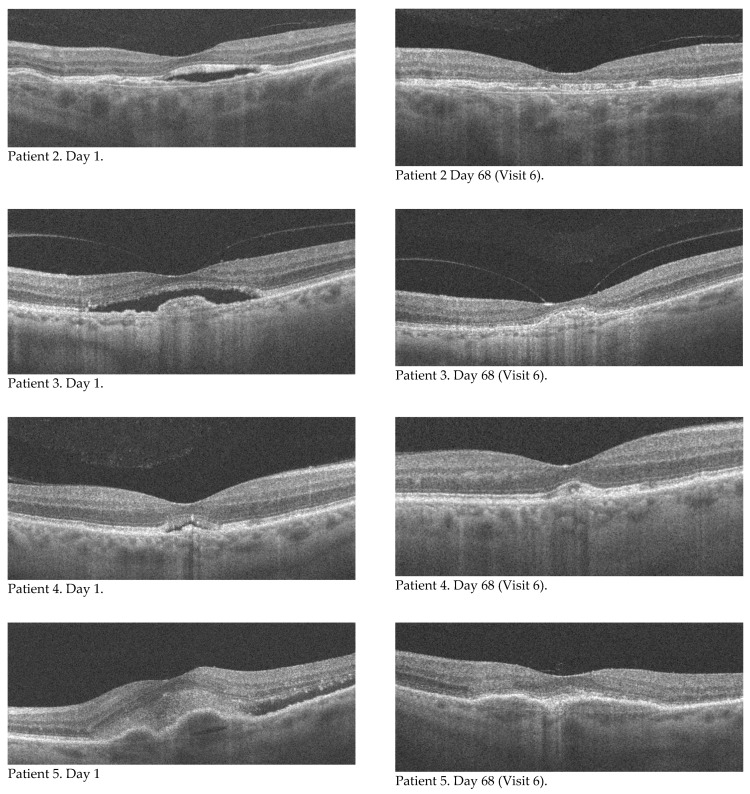

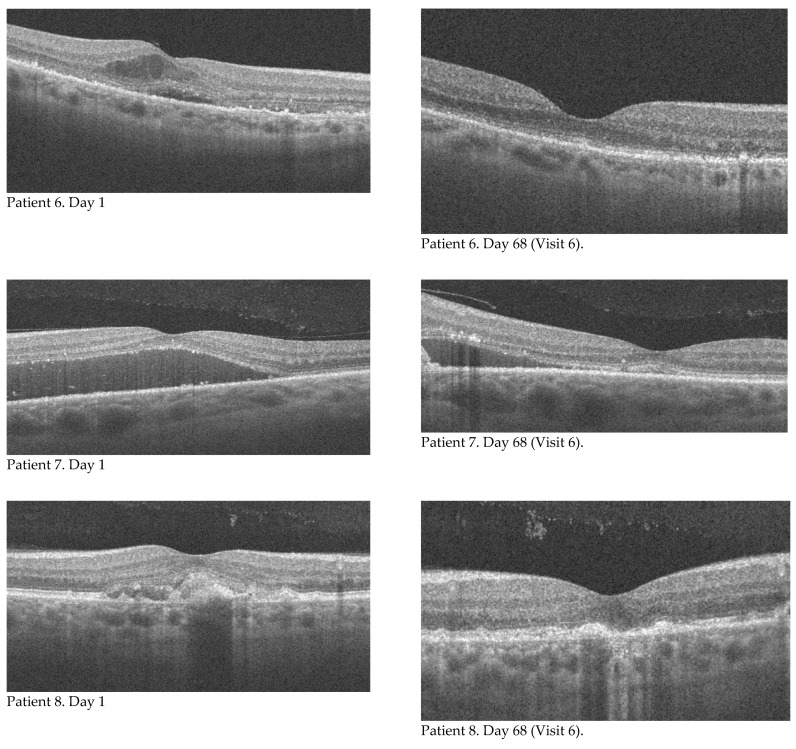
Changes in BCVA in subsequent measurements. Colour indicates statistically significant differences between two consecutive measurements. The change in central retinal morphology as seen on OCT (B-scan) in individual patients on day 1 of injection and day 97 (endpoint) is shown above.

The mean value of best corrected visual acuity (BCVA) increased with each visit. The results recorded in the study group along with the standard deviation value are shown in Table 2. The Student’s *t* test for dependent samples showed a significant difference already between the baseline value (BCVA_1) and the measurement taken seven days after the first injection at the follow-up visit (BCVA_2). This relationship and the changes in BCVA in subsequent measurements are shown in Figure 1. The value of best corrected visual acuity continued to increase at subsequent measurement points, but the differences between successive results were not statistically significant. The total change in BCVA during the course of the therapy was also analyzed by comparing the baseline value (BCVA_1) and the result of the last follow-up visit, that is, seven days after the third injection (BCVA_6). The mean change in the studied group was 14.5 letters (SD = 9.61). There was no improvement in one patient (the final value corresponded to the baseline value). This was most likely related to the presence of focal sub foveal photoreceptor atrophy, already present at the time of treatment initiation. An improvement in BCVA ranging from 9 to 30 letters.

**Figure 2:**
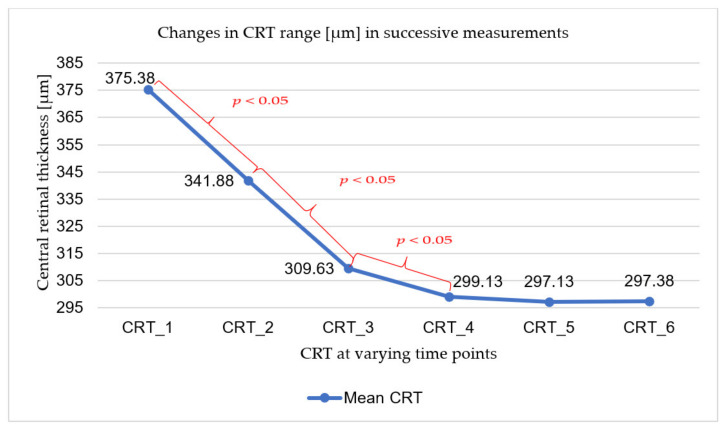
Changes in CRT range (µm) in successive measurements. Color indicates statistically significant differences between two consecutive measurements.

The mean central retinal thickness (CRT) outcome decreased with each subsequent visit, except for the last measurement. The mean central retinal thickness results achieved in the study group along with the standard deviation values are shown in Table 3. Analyses of the subsequent CRT measurements showed a significant difference as early as between the first (CRT_1—baseline value on the day of the first injection) and the second measurement (CRT_2—follow-up seven days after the first injection) and between the two subsequent measurements (CRT_2 and CRT_3 and CRT_3 and CRT_4). At the next measurement (CRT_5), the CRT value also decreased, but compared to the result at the previous visit (CRT_4), this change was not statistically significant. At the last checkpoint (CRT_6), the mean CRT value had a non-statistically significant increase to 297.38. The above relationships are presented in Figure 2.

The total change in central retinal thickness during the course of the therapy was also analysed, comparing the result achieved in the first (CRT_1) and last measurement (CRT_6). Each subject had a decrease in retinal thickness, with a mean change of 79 um (SD = 65.44; min.-max: 19–226).

Regarding the neovascular membrane area, a statistically significant change was shown after the first injection (difference between baseline and measurement at the follow-up visit seven days after the first injection). During further therapy, the area of the neovascular membrane underwent statistically insignificant changes. It initially increased (between the second and third measurements) and then decreased up to and including the last measurement. The mean changes in neovascular membrane area along with the standard deviation value and the results of significance tests for differences between successive measurements are presented in Table 4.

**Figure 3:**
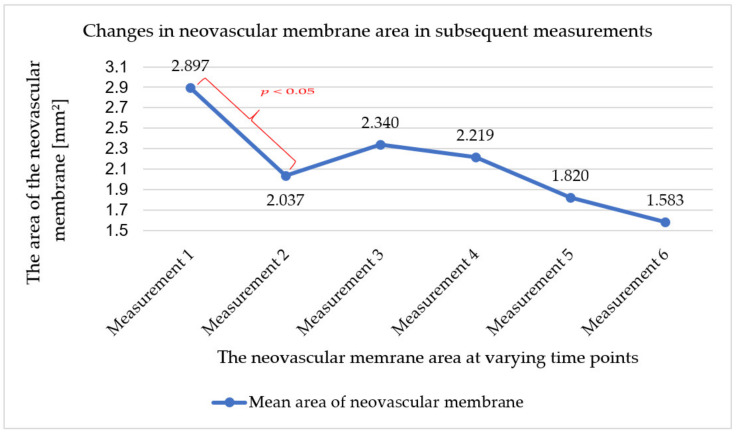
Changes in mean neovascular membrane area in subsequent measurements. Color indicates statistically significant differences between two consecutive measurements.

The total change of the neovascular membrane area in the course of the applied therapy was also analysed, comparing the result obtained in the first and last measurement. These relationships are shown in Figure 3. The mean change in the area of the neovascular membrane in each patient was −1.32 mm^2^ (SD = 1.21; min.-max.: 0.05–3.02).

**Figure 4:**
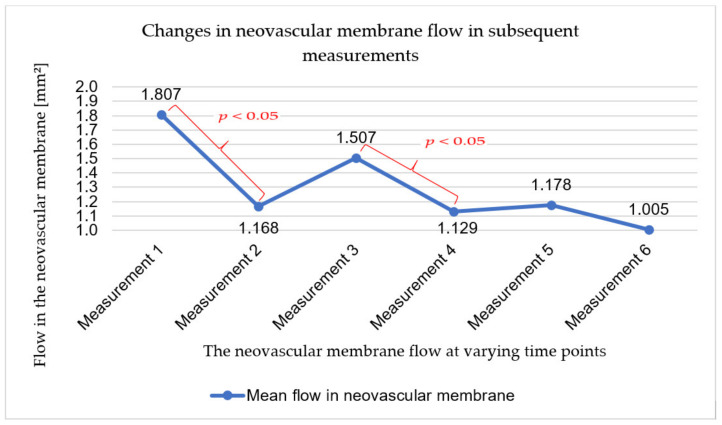
Changes in neovascular membrane flow in subsequent measurements. Color indicates statistically significant differences between two consecutive measurements.

With regard to flow in the neovascular membrane, a statistically significant change was demonstrated after the first injection (between the first and second measurements) and after the second injection (between the third and fourth measurements). Beyond these control points, the flow in the neovascular membrane underwent statistically insignificant changes. It increased (between the second and third measurements and between the fourth and fifth measurements) and decreased after the third injection (between the fifth and sixth measurements). These relationships are shown in Figure 4. The mean changes in flow in the neovascular membrane along with the standard deviation value and the results of significance tests for differences between successive measurements are presented in Table 5. 

The total change of flow in the neovascular membrane during the course of therapy was also analyzed by comparing the results obtained in the first and last measurements. Each patient had a decrease in neovascular membrane flow. The mean change in the studied group was −0.802 mm^2^ (SD = 0.718; min–max: 0.09–1.76).

In addition, for the results obtained in the first measurement (baseline, on the day of the first injection), no significant correlations were found except for two variables concerning the neovascular membrane. The area of the neovascular membrane was strongly positively correlated with the flow in this membrane, and this relationship was statistically significant (r = 0.998; *p* < 0.01). A similar relationship was found in the correlation analysis of variables determining the difference between the first and last measurements. There was also a strong positive correlation between area and flow in the choroidal membrane. This relationship was statistically significant (r = 0.990; *p* < 0.01).

## 5. Discussion

Brolucizumab is an innovative anti-VEGF molecule that has been used to treat the exudative form of age-related macular degeneration (wAMD). It is effective in reducing all types of fluid that manifest as disease activity: subretinal fluid, intraretinal fluid, and fluid under the pigment epithelium (RPE) [7]. Therapy with this molecule can restore the anatomical image of the retina and improve visual acuity.

Brolucizumab is a single-chain monoclonal antibody fragment of the mass of only 26 kDa, smaller than aflibercept (97–115 kDa) or ranibizumab (48 kDa). This allows for a higher molar concentration (12 x higher compared to aflibercept) and consequently better penetration of retinal structures compared to aflibercept and ranibizumab [8].

The low molecular weight and higher molar concentration also provide more active molecules of the drug that neutralize the VEGF mediator. The absence of the Fc fragment is also an advantage from a pharmacokinetic standpoint [9]. Results from phase I and phase II clinical trials have confirmed the efficacy and longer-lasting effect of brolucizumab in the treatment of wAMD [10]. In the pivotal phase III studies HAWK and HARRIER, involving a total of 1817 patients, brolucizumab administered in some patients every 3 months after the saturation phase reduced disease activity successfully. A better therapeutic effect was achieved with fewer injections vs. aflibercept, which provides an opportunity to reduce the treatment burden of patients with wAMD [9].

The HAWK and HARRIER trials demonstrated the high therapeutic efficacy of brolucizumab, confirmed by disease regression scores. Patients treated with brolucizumab had approximately 30% less disease activity compared with patients treated with aflibercept, which confirmed the overall good safety profile of this therapy [7].

A rare complication to be aware of is intraocular inflammation, including retinal vasculitis, which may be accompanied by vascular occlusion. A case report of a 76-year-old Caucasian woman who developed uveitis and vitreoretinitis one week after receiving her third monthly injection of brolucizumab into the vitreous body [11]. Improvement was achieved after topical application of 1% prednisolone acetate drops. One month after the last brolucizumab injection, the patient was given ranibizumab. Three weeks after the ranibizumab injection, the patient again developed symptoms of inflammation, both intraocular and retinal vasculitis with accompanying vascular occlusion, with significant deterioration of visual acuity. The patient underwent vitrectomy through the pars plana and biopsy of the vitreous body for microbiological and cytopathological studies. The results of these studies showed a chronic inflammatory infiltrate without features of bacterial infection. Treatment with brolucizumab may therefore cause intraocular inflammation and retinal vasculitis in rare cases, probably due to a delayed hypersensitivity reaction to the drug. In conclusion, it should be emphasized that the safety profile of brolucizumab is good.

In another publication, the authors report the response to brolucizumab injected into the vitreous body in patients previously treated with anti-VEGF therapy for wAMD [12]. Six eyes (six patients) with visual deterioration or no improvement during treatment with the anti-VEGF drugs aflibercept or bevacizumab were studied. After 4 weeks of brolucizumab administration, improvements were observed in IRF/SRF reduction, central retinal thickness, and mean perifoveal thickness in the optical coherence tomography (OCT) scans performed. No serious adverse effects were observed, including signs of vasculitis or increased cell counts in the anterior chamber. It was concluded that brolucizumab is a safe and effective therapeutic option for patients with refractory CNV in exudative AMD.

## 6. Conclusions

Our study confirmed the efficacy of brolucizumab in treating patients with exudative AMD in terms of improvements in best corrected visual acuity (BCVA), central retinal thickness (CRT), neovascular membrane area (NVMA), and neovascular membrane flow area (NVMAF). Our observations of improved BCVA and reduced CRT as a result of brolucizumab therapy are consistent with the noninferior results observed in registration studies of the most recent anti-VEGF drug [7].

The Hawk and Harrier study also reported progressive improvements in BCVA and CRT after each of the first three injections. Follow-up measurements were performed on the day of the first injection and then at 4-week intervals. In our study, we used a different and innovative technique to assess the efficacy of brolucizumab, namely on the day of each of the first three injections and then as early as 1 week after each injection. As early as day 7 after the first injection, we obtained statistically significant improvement in BCVA, reduction in CRT, reduction in NVMA, and reduction in NVMAF. The Hawk and Harrier registration studies did not evaluate neovascular membrane area or neovascular membrane flow area. In the literature on the efficacy of brolucizumab in the treatment of exudative AMD, we did not find such observations, nor did we find reports describing the regime we introduced for the assessment of individual parameters. The trend of improvement in these parameters was maintained throughout the study. There was also a statistically significant positive correlation between the reduction of NVMA and NVMAF. No adverse effects, either local or systemic, were observed among the patients included in our study.

## Figures and Tables

**Table 1:** Main variables in the study—baseline characteristics (values at first measurement).

	Mean (M)	Standard Deviation (*SD*)	Minimum (Min.)	Maximum (Max.)
Age (*n* = 8)	71 years and 3 months	8,86	59	84
Male (*n* = 5)	72	7.35	62	80
Female (*n* = 3)	70	12.77	59	84
Best corrected visual acuity of the examined eye *(BCVA)*	59.31	14.27	35	77.5
Visual acuity of the examined eye (according to the Snellen chart) *(VA)*	0.36	0.21	0.1	0.7
Central retinal thickness in µm (*CRT*)	375.38	80.04	308	564
Area of the neovascular membrane [mm^2^]	2.9	2.92	0.389	9.047
Flow in the neovascular membrane [mm^2^]	1.81	1.83	0.224	5.749

**Table 2:** BCVA—Descriptive statistics and statistical significance of differences.

	BCVA—descriptive statistics					
	BCVA_1	BCVA_2	BCVA_3	BCVA_4	BCVA_5	BCVA_6
Mean result (*M*)	59.313	66.625	66.813	70.563	73.063	73.813
Standard deviation (*SD*)	14.27	10.78	12.02	11.69	12.45	12.69
	BCVA—statistical significance of differences between consecutive measurements ^a^					
	BCVA_1 and BCVA_2	BCVA_2 and BCVA_3	BCVA_3 and BCVA_4	BCVA_4 and BCVA_5	BCVA_5 and BCVA_6	
Mean difference (*M*)	7.31	2.19	1.75	2.5	0.75	
Standard deviation (*SD*)	4.53	3.12	7.31	4.66	2.12	
*t*(7)	2.375	1.633	0.730	1.342	1.000	
Relevance (bilateral)	0.018	0.102	0.465	0.180	0.317	

Statistically significant differences between consecutive measurements are marked in color. ^a^ Student’s *t*-test for dependent samples.

**Table 3:** CRT—descriptive statistics and statistical significance of differences.

	CRT (µm) in subsequent measurements—descriptive statistics					
	CRT_1	CRT_2	CRT_3	CRT_4	CRT_5	CRT_6
Mean result (*M*)	375.38	341.88	309.63	299.13	297.13	297.38
Standard deviation (*SD*)	80.04	66.69	28.01	23.84	24.12	25.19
	CRT—descriptive statistics and statistical significance differences between consecutive measurements ^a,b^					
	CRT_1 i CRT_2	CRT_2 i CRT_3	CRT_3 i CRT_4	CRT_4 i CRT_5	CRT_5 i CRT_6	
Mean difference (*M*)	−33.50	−32.25	−10.50	−2.00	0.25	
Standard deviation (*SD*)	27.84	44.54	11.19	5.37	2.05	
Test statistics	−2.521 ^a^	−2.524 ^a^	−2.655 ^b^	−1.053 ^b^	0.344 ^b^	
Relevance (bilateral)	0.012	0.012	0.033	0.327	0.741	

Statistically significant differences between consecutive measurements are marked in color. ^a^ Wilcoxon test—Z statistic. ^b^ Student’s *t*-test for dependent samples—*t*(7) statistic.

**Table 4:** Neovascular membrane area-descriptive statistics and statistical significance of differences.

	Neovascular membrane area (mm^2^) in consecutive measurements—descriptive statistics					
	Measurement 1	Measurement 2	Measurement 3	Measurement 4	Measurement 5	Measurement 6
Mean result (*M*)	2.897	2.037	2.34	2.219	1.82	1.583
Standard deviation (*SD*)	2.92	1.95	2.99	2.33	2.2	1.92
	Neovascular membrane area - descriptive statistics statistical significance of differences between successive measurements ^a,b^					
	Measurement 1 and 2	Measurement 2 and 3	Measurement 3 and 4	Measurement 4 and 5	Measurement 5 and 6	
Mean difference (*M*)	−0.861	0.303	−0.121	−0.399	−0.237	
Standard deviation (*SD*)	1.02	1.25	1.2	0,97	0.34	
Test statistics	−2.392 ^a^	0.420 ^b^	−1.4 ^b^	−0.845 ^b^	−1.960 ^b^	
Relevancy (bilateral)	0.048	0.674	0.161	0.398	0.050	

Statistically significant differences between consecutive measurements are marked in color. ^a^ Student’s *t*-test for dependent samples—*t*(7) statistic. ^b^ Wilcoxon test—Z statistic.

**Table 5:** Flow in the neovascular membrane-descriptive statistics and statistical significance of differences.

	Flow in the neovascular membrane (mm^2^) in successive measurements—descriptive statistics					
	Measurement 1	Measurement 2	Measurement 3	Measurement 4	Measurement 5	Measurement 6
Mean result (*M*)	1.807	1.168	1.507	1.129	1.178	1.005
Standard deviation (*SD*)	1.83	1.23	2.01	1.37	1.50	1.28
	Flow in the neovascular membrane - descriptive statistics statistical significance of differences between consecutive measurements ^a^					
	Measurement 1 and 2	Measurement 2 and 3	Measurement 3 and 4	Measurement 4 and 5	Measurement 5 and 6	
Mean difference (*M*)	−0.64	0.339	−0.378	0.049	−0.172	
Standard deviation (*SD*)	0.71	0.83	0.65	0.2	0.24	
Test statictics	−2.521	0.700	−2.521	0.676	−2.240	
Relevancy (bilateral)	0.012	0.484	0.012	0.499	0.085	

Statistically significant differences between consecutive measurements are marked in color. ^a^ Wilcoxon test—Z statistic.

## Data Availability

Data are contained within the article.
